# Stiff Knee Gait After Stroke: The Potential Compensatory Role of Mid-Swing Rectus Femoris Activity

**DOI:** 10.1155/crnm/7703081

**Published:** 2025-10-03

**Authors:** Wieneke van Oorschot, Bente Bloks, Jip Kamphuis, Allan Pieterse, Alexander Geurts, Noël Keijsers, Jorik Nonnekes

**Affiliations:** ^1^Department of Rehabilitation, Sint Maartenskliniek, Nijmegen, the Netherlands; ^2^Department of Research, Sint Maartenskliniek, Nijmegen, the Netherlands; ^3^Department of Rehabilitation, Radboud University Medical Centre, Nijmegen, the Netherlands; ^4^Department of Sensorimotor Neuroscience, Donders Institute for Brain, Cognition and Behaviour, Radboud University, Nijmegen, the Netherlands

**Keywords:** rectus femoris, spasticity, stiff knee gait, stroke, upper motor neuron disease

## Abstract

Reduced knee flexion during the swing phase of gait, commonly referred to as ‘stiff knee gait,' is frequently encountered in patients with upper motor neuron syndrome, e.g., due to stroke. Rectus femoris spasticity is one of the main causes of stiff knee gait and can be treated with botulinum toxin (BoNT-A) injections. However, previous literature shows large response variations after BoNT-A treatment between individual participants. These variations could be due to the overestimation of rectus femoris spasticity during gait analyses based on the current main indicator: mid-swing rectus femoris activity. The objective of this video-illustrated case series, including gait data of four stroke patients with stiff knee gait, is to propose an alternative explanation for this mid-swing rectus femoris activity. The presented patients all show mid-swing rectus femoris activity, which could be considered a sign of spasticity but was interpreted as compensatory activity to improve foot clearance during the swing phase instead. Misinterpretation of compensatory rectus femoris activity as spasticity may lead to inadequate treatment with BoNT-A injections in some patients, possibly explaining the response variations found in previous literature. Additional biomechanical markers should be explored to better determine the contribution of rectus femoris spasticity in stiff knee in individual patients.

## 1. Introduction

People with upper motor neuron syndrome (e.g., due to stroke) often show reduced knee flexion during the swing phase of gait, commonly referred to as ‘stiff knee gait' [1–4]. Stiff knee gait impairs foot clearance of the affected leg, thereby increasing fall risk and energy costs of walking. In unaffected gait, knee flexion during the swing phase is not initiated by the knee flexors. Instead, during preswing, push-off from the calf muscles in combination with pull-off from the large hip flexors is responsible for an extrinsic knee flexion moment, forcing the knee into rapid flexion. The rectus femoris muscle, a biarticular muscle that crosses the hip and knee joints, regulates the degree of knee flexion during the swing phase through eccentric contraction, causing a counterbalancing intrinsic knee extension moment [5–7]. Without rectus femoris activity, excessive knee flexion would occur, whereas rectus femoris overactivity results in reduced knee flexion.

There are roughly three factors that contribute to stiff knee gait in people with upper motor neuron syndrome, either individually or in combination. First, decreased push-off at the ankle joint may lead to a lower extrinsic knee flexion moment during preswing, leading to a lower swing-phase knee flexion angle [1–4, 6–13]. Second, decreased pull-off at the hip joint may also cause a lower extrinsic knee flexion moment during preswing, resulting in less swing-phase knee flexion [4, 8–10, 12]. Third, inappropriate activity of the quadriceps femoris, including the rectus femoris, potentially causes a premature deceleration of the knee flexion by generating an intrinsic knee extension moment [1–10, 12].

The above-mentioned causal triad of stiff knee gait is not easily disentangled in individual patients, as it requires a three-dimensional (3D) instrumented gait analysis [14]. Clinical literature seems to focus on rectus femoris spasticity as the main cause of stiff knee gait. Such spasticity can be reduced by chemodenervation of the muscle, for example with botulinum toxin (BoNT-A) injections [1, 4, 8]. However, previous studies on the effect of BoNT-A injections on stiff knee gait have generated inconsistent results, with large variations between individual study participants and often rather small improvements in swing phase knee flexion angle after treatment [2–4, 8, 13, 15].

The indication for treatment with BoNT-A is typically based on the presence of reduced knee flexion during the swing phase of gait—frequently characterized by a distinctive interruption of the knee flexion kinematic curve (i.e., a ‘double bump shape') [13, 15]—combined with inappropriately timed rectus femoris activity measured with surface electromyography (sEMG) [13]. Notably, interpreting rectus femoris activity during the gait cycle is not straightforward. Theoretically, rectus femoris spasticity would be triggered during preswing, when knee flexion angular velocity peaks. Yet, it can be difficult to label preswing sEMG activity as inappropriate, as in unaffected walking the rectus femoris should become active during preswing to decelerate knee flexion [5, 12]. Therefore, the presence of mid-swing rectus femoris activity is often used as a criterion for rectus femoris spasticity during gait [2–4, 8, 16].

The objective of this video-illustrated case series is to question the use of mid-swing rectus femoris activity as a criterion of rectus femoris spasticity and therefore also as an indication for treatment with BoNT-A injections. This notion is substantiated based on four patients with stiff knee gait after stroke who were referred to our hospital for a clinical 3D instrumented gait analysis.

## 2. Case Presentation

**Figure 1:**
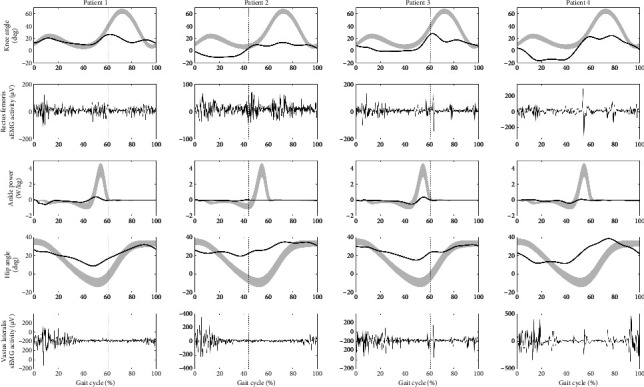
[Gait characteristics of a full gait cycle of four cases with chronic stroke and stiff knee gait.]. The figure includes knee kinematics, rectus femoris sEMG activity, ankle power, hip kinematics, and vastus lateralis sEMG activity during the gait cycle (initial contact–initial contact). Norm data for kinematics and ankle power is shown in grey. The vertical dotted line indicates the transition from stance phase to swing phase. Please be aware of the individually scaled sEMG graphs and of possible motion artifacts in the sEMG data at preswing and initial swing in patients 3 and 4.

This video-illustrated case series presents four patients (ages 41 to 56), who were in the chronic phase after stroke (16 to 64 months) and referred to our hospital for a clinical 3D gait analysis.  Figure 1 shows the knee flexion angle, rectus femoris sEMG, ankle power, hip flexion angle, and vastus lateralis sEMG for all patients separately. Gait speed varied between 0.36 and 0.64 m/s.

### 2.1. Diagnostic Assessment

All four patients (see Figure 1 and Videos [Supporting Information 1–4]) displayed a reduced peak knee flexion angle in the swing phase (13.3–27.5°), with the presence of a distinct interruption of knee flexion in their knee kinematic curve, the so called ‘double bump'. They all suffered from severe weakness of the calf muscles on the affected side (Medical Research Council [MRC] scale scores 0–2) upon clinical examination and strongly diminished or even absent push-off power at the ankle joint during walking, as assessed with force plates. Furthermore, they all had moderate weakness of the hip flexors (MRC scores 3–4). Finally, all showed mid-swing rectus femoris activity, which could be interpreted as rectus femoris spasticity, and all showed spasticity of the rectus femoris during clinical examination (Tardieu scale [V2] scores 1–3). Relevant individual results of the strength and spasticity tests are presented in Table 1.

### 2.2. Clinical Reasoning

Based on the presence of stiff knee gait, the clinical examination, and mid-swing rectus femoris activity, one would usually conclude that rectus femoris spasticity played a role in the stiff knee gait in these patients and therefore proceed with spasticity treatment. However, closer examination of the rectus femoris sEMG data, aligning the timing of the mid-swing rectus femoris activity with the knee and hip kinematics, suggested an alternative explanation of the mid-swing rectus femoris activity for two reasons. First, rectus femoris activity occurred simultaneously with or prior to an acceleration in knee flexion, whereas in the case of spasticity, one would expect such activity to coincide with a subsequent deceleration of knee flexion. Second, the rectus femoris activity occurred simultaneously with a flexion motion in the hip. Therefore, mid-swing rectus femoris activity may not just be inappropriately timed activity due to spasticity but could also reflect compensation through hip flexion in an effort to improve foot clearance, threatened by a severely decreased push-off.

**Figure 2:**
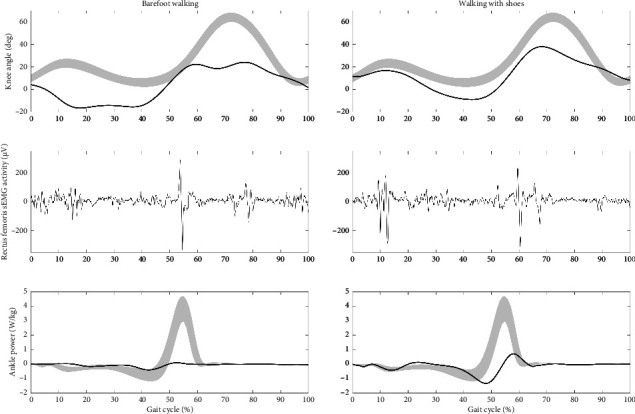
[Knee kinematics, rectus femoris sEMG activity, and ankle power of patient 4 during the gait cycle (initial contact–initial contact) for two gait conditions: barefoot walking and walking with orthopedic footwear and a dorsal anklefoot orthosis]. Norm data for kinematics and ankle power is shown in grey. The vertical dotted line indicates the transition from stance phase to swing phase. Please be aware of possible motion artifacts in the sEMG data at preswing in both conditions.

This possible compensatory nature of rectus femoris activity is supported by the differences between barefoot walking (see Video, Supporting Information 4) versus walking with orthopedic footwear and a dorsal ankle-foot orthosis (see Video, Supporting Information 5) in patient 4.  Figure 2 presents the individual's knee kinematics, rectus femoris sEMG activity, and push-off power in both conditions. When walking barefoot, the patient showed diminished and interrupted swing-phase knee flexion, decreased push-off power, and mid-swing rectus femoris activity. When walking with orthopedic footwear and a dorsal ankle-foot orthosis, ankle push-off power improved, leading to an increase in walking speed from 0.50 to 0.78 m/s. Furthermore, maximum knee flexion in the swing phase improved from 24° to 36°, preswing knee flexion angular velocity from 239°/s to 332°/s, and the interruption in the knee flexion kinematic curve disappeared. Remarkably, the rectus femoris overactivity during mid-swing completely disappeared when walking with orthopedic footwear. In the case of rectus femoris spasticity, one would expect a higher walking speed and knee flexion angular velocity to elicit a stronger velocity-dependent stretch reflex of the rectus femoris during preswing. Given that the opposite occurred while walking with orthopedic footwear, the swing phase knee flexion likely improved due to the higher push-off power. This was probably due to prior improvement of the second ankle rocker in mid-stance with the AFO-footwear combination. The mid-swing rectus femoris activity in the barefoot condition was therefore probably a compensation for the lack of ankle push-off and not an expression of spasticity. This conclusion is in line with a recent study in healthy individuals that showed that rectus femoris activity during the swing phase increased following a series of fatiguing, repetitive concentric hip flexion exercises performed until task failure [16].

## 3. Discussion

It is fair to conclude that determining the contribution of rectus femoris spasticity to stiff knee gait in people with upper motor neuron syndrome is complex and that interpretation of rectus femoris sEMG activity is difficult [17]. The observations described in this case series indicate that the timing of rectus femoris activity in relation to knee and hip kinematics, and thus the potential compensatory role of the rectus femoris, should be considered during clinical interpretation of 3D gait analysis data. This is especially relevant for those with a strongly diminished push-off, e.g., due to calf weakness, abnormal second rocker, pain, abnormal structure of the ankle/foot complex, lack of selective motor control, or a combination of these factors. Furthermore, when interpreting rectus femoris activity, walking speed should be taken into account. When walking at low velocities, the need for regulation of knee flexion by the rectus femoris is much lower [18]. Misinterpretation of compensatory activity of the rectus femoris as spasticity could lead to a suboptimal treatment of stiff knee gait. Furthermore, our observations may at least partly explain the large response variations in several studies examining the effect of BoNT-A on stiff knee gait when mid-swing rectus femoris activity is used as an inclusion criterion in these studies [2, 3, 15]. For example, one of these studies by Tenniglo et al. presented a knee kinematic curve of a patient with little treatment effect and, although ankle kinetics were not shown, this patient exhibited knee hyperextension during the stance phase, probably hampering effective push-off [3]. This patient may have had a substantially decreased push-off that could have been the primary cause of the stiff knee gait instead of rectus femoris spasticity.

Hence, there is a need for a better marker to determine the contribution of rectus femoris spasticity to stiff knee gait. Interestingly, a study by Campanini and colleagues described a promising method to determine the underlying cause of stiff knee gait based on the linear relationship between maximum knee flexion angular velocity and the vertical acceleration of the lateral malleolus during preswing [9]. This relationship was assessed in healthy individuals walking at several speeds, also comparing this data with that obtained from participants with stiff knee gait in the chronic phase of stroke. Of the 52 included participants with stiff knee gait, 85% fell within the 95% confidence interval of the regression model, suggesting that decreased push-off was the primary cause of their stiff knee gait. Yet, three participants with a lower knee flexion velocity than expected based on the regression model were suspected of rectus femoris spasticity and treated with a diagnostic femoral nerve block (motor branch of rectus femoris) using a local anesthetic (lidocaine). This block resulted in varying increments in peak knee flexion of 2°, 10°, and 30°, corresponding with the magnitude of their deviation from the model. A recent study by our team and colleagues presented a more comprehensive model that included both ankle push-off and hip pull-off and explained a significant amount of peak knee flexion in patients after stroke [19]. Biomechanical measures of push-off and pull-off in relation to maximum knee flexion angular velocity could be useful clinical markers to better estimate the contribution of quadriceps femoris spasticity to stiff knee gait [20]. In conclusion, misinterpretation of compensatory mid-swing rectus femoris activity as spasticity could possibly lead to inadequate treatment with BoNT-A injections in some patients and may explain response variations to BoNT-A treatment found in previous literature. Further research in this field is required to improve the diagnostic process and to better tailor treatment strategies for individuals with stiff knee gait.

## Figures and Tables

**Table 1:** Strength and spasticity measures from clinical physical examination.

	Patient 1	Patient 2	Patient 3	Patient 4
Muscle strength affected side (MRC 0–5)				
Hip flexors	4	4	4	3
Knee extensors	4	4	5	4
Ankle dorsiflexors	0	1	3	1
Ankle plantar flexors	1	0	2	0
Spasticity affected side (MAS 0–4)				
Gastrocnemius	0	1	1	2
Soleus	0	1	1	1
Rectus femoris	0	3	1	1
Spasticity affected side (tardieu [V2] scale 0–4)				
Gastrocnemius	0	1	2	2
Soleus	0	2	1	4
Rectus femoris	1	3	2	1

Abbreviation: MAS, Modified Ashworth Scale.

## Data Availability

The data that support the findings of this study are available on request from the corresponding author. The data are not publicly available due to privacy or ethical restrictions.

## Supporting Information

Supporting Information 1 Supporting Information 1: A 46-year-old patient with a right-sided hemiparesis after an ischemic stroke (2 years and 3 months ago). This video corresponds with the graphs presented for patient 1 in Figure 1.

Supporting Information 2 Supporting Information 2: A 51-year-old patient with a right-sided hemiparesis after an ischemic stroke (2 years ago). The patient was not able to walk barefoot without support and is therefore shown while walking with store-bought footwear. This video corresponds with the graphs presented for patient 2 in Figure 1.

Supporting Information 3 Supporting Information 3: A 56-year-old patient with a left-sided hemiparesis after a hemorrhagic stroke (1 year and 5 months ago). This video corresponds with the graphs presented for patient 3 in Figure 1.

Supporting Information 4 Supporting Information 4: A 41-year-old patient with a right-sided hemiparesis after an ischemic stroke (6 years ago). This video corresponds with the graphs presented for patient 4 in Figure 1, and the graphs presented for barefoot walking in Figure 2.

Supporting Information 5 Supporting Information 5: Patient 4, the same patient as Supporting Information 4 (41-year-old patient with a right-sided hemiparesis after an ischemic stroke (6 years ago), walking with orthopedic shoes and a dorsal ankle-foot-orthosis. This video corresponds with the graphs presented for shoe-walking in Figure 2.
